# Light-emitting diode fluorescent microscopy and Xpert MTB/RIF® assay for diagnosis of pulmonary tuberculosis among patients attending Ambo hospital, west-central Ethiopia

**DOI:** 10.1186/s12879-017-2701-5

**Published:** 2017-09-11

**Authors:** Alemu Gadissa Gelalcha, Abebaw Kebede, Hassen Mamo

**Affiliations:** 1 Ambo Hospital, west-central, Ambo, Ethiopia; 2 Ethiopian Public Health Institute, Federal Ministry of Health, Addis Ababa, Ethiopia; 3 Department of Microbial, Cellular and Molecular Biology, College of Natural Sciences, Addis Ababa University, P O Box, 1176 Addis Ababa, Ethiopia

## Abstract

**Background:**

The relatively simple and cheaper light-emitting diode fluorescent microscopy (LED-FM) was recommended by the World Health Organization (WHO) to replace the conventional tuberculosis (TB) microscopy in both high- and low-volume laboratories. More recently the WHO also endorsed one more technique, Xpert MTB/RIF® assay (Xpert), for improved TB diagnosis particularly among human immunodeficiency virus (HIV)-infected cases. However, the relative performance of both of these tools differs from setting to setting in reference to the conventional TB diagnostics. This study thus aimed to evaluate these tools for TB detection in individuals visiting Ambo Hospital, west-central Ethiopia.

**Methods:**

Cross-sectional early-morning sputum samples were collected from presumptive TB patients between January and August 2015. Socio-demographic data were captured using a structured questionnaire. Clinical information was gathered from patients’ medical records. The sputum samples were diagnosed using LED-FM, Xpert, concentrated Ziehl-Neelsen (cZN) staining and Lowenstein-Jensen (LJ) culture as the gold standard. Drug sensitivity test (DST) was also conducted.

**Results:**

Out of 362 sputum samples collected and processed, 36(9.9%) were positive by LED-FM, 42(11.6%) by cZN and 50(13.8%) by Xpert. But, only 340 samples could be declared culture positive or negative for mycobacteria. Of these 340, eight were non-tubercle mycobacteria (NTM). Out of the remaining 332 samples, 45(13.6%) had culture-confirmed TB with 11(24.4%) being HIV co-infected. LED-FM, Xpert and culture detected 54.5% (6/11), 90.9% (10/11) and 100% (11/11) mycobacteria in HIV-positive individuals and 81.3% (26/32), 73.7% (28/38), 78.8% (26/33) and 73.2% (30/41), in HIV negatives respectively. Two samples were rifampicin resistant by both Xpert and DST. The overall sensitivity, specificity, positive and negative predictive values of LED-FM and Xpert were 77.8, 100, 100 and 96; and 93.3, 98, 97.5 and 98.9% respectively.

**Conclusion:**

The data demonstrated the high diagnostic yield of Xpert. LED-FM sensitivity is higher compared to results quoted by recent systematic reviews although it appears to be lower than what was cited in the WHO policy statement (83.6%) during the recommendation of the technology. The high specificity of LED-FM in the study area is encouraging and is expected to boost its reliability and uptake.

## Background

Tuberculosis (TB) is caused by bacteria belonging mainly to *Mycobacterium tuberculosis* complex (MTBC). Among the species of MTBC, *M. tuberculosis* (MTB) is the dominant cause of TB. MTB is the most common opportunistic infection in human immunodeficiency virus (HIV)-infected individuals and a cause of HIV-related deaths. Although the number of TB-related deaths dropped by 22% in the last 15 years (2000–2015), TB remained among the leading (top-ten) causes of global fatality in 2015 with about 1.4 million deaths and an additional 0.4 million deaths among co-infected people with HIV [1].

One of the most important reasons for the high number of TB-related deaths in low-income countries is the difficulty in diagnosis. Conventional light microscopy (LM) screening of Ziehl-Neelsen (ZN) stained sputum smears is still the mainstay of pulmonary TB (PTB) diagnosis in rural settings despite its lower sensitive especially in HIV-positive patients, extrapulmonary TB cases, children and patients with paucibacillary nature in general [2, 3].

Light-emitting diode fluorescent microscopy (LED-FM) which uses auramine-stained smear techniques is simpler, cheaper and having a longer lifespan. It does not produce ultraviolet light, has minimal power requirements and better performance, on average 10% more sensitive than conventional ZN-LM [2, 4–7]. Additional advantages of this technology is that lower magnifications can be used, enabling rapid screening over a wider area of the smear to be seen and resulting in up to 4 times faster examination of smear, its lower maintenance requirements and ability to run on batteries [8, 9]. This technology is believed to be more beneficial in TB high-burden and resource-limited settings. As a result, the World Health Organization (WHO) recommended the introduction of LED-FM to replace LM in both high- and low-volume laboratories [7]. The WHO also recently endorsed one more technique, Xpert MTB/RIF® assay (Xpert), for better TB diagnosis particularly among HIV-infected cases.

However, the relative performance of the two tools differs from setting to setting in reference to the gold standard mycobacterium culture method. Especially, the uptake of LED-FM is low because of its reported or perceived low specificity. In 2013, only 6% of microscopy centres had reportedly replaced ZN by LED-FM [10]. This study was, thus, aimed at evaluating these tools for TB diagnosis in individuals visiting Ambo Hospital, west-central Ethiopia.

Ethiopia is in the WHO list of the 20 TB high-burden countries [1]. Moreover, the country is among TB/HIV high-burden countries with approximately 10% co-infection rate and TB case detection rate is 60%, which is below the target (72%) [11]. So, Ethiopia should increase its TB case finding. Periodic evaluation of TB diagnosing tools is one method for maximizing TB detection rate in the country and better control the disease.

The study generated new data about the diagnostic performance of LED-FM and Xpert MTB/RIF® assay in presumptive TB patients at Ambo Hospital. The results have implications for policymakers in scaling-up implementation of both technologies in the national TB diagnosis algorithm.

## Methods

### Study site and design

Health-facility-based cross-sectional study was conducted on patients with presumptive TB to evaluate the performance of LED-FM and Xpert assay for detection of MTBC. The study was conducted at Ambo Hospital in Ambo town which is 112 km in the west of Addis Ababa, the Ethiopia capital.

### Study participants

All TB presumptive patients aged ≥5 years and who provided sufficient sputum samples were recruited. Both new and those with previous history of TB medication, and multi-drug resistant TB (MDR-TB) suspected patients including HIV-infected and non-infected cases were eligible for inclusion in the study. Patients who were on anti-TB medication (follow-up patients), and those with severe illness were excluded. Patients were recruited prospectively in a consecutive manner until the estimated sample size of 362 was obtained. The study was conducted from January through August 2015.

### Sample collection and analysis

A structured questionnaire with slight modification from that prepared by the WHO [12] was administered to capture socio-demographic and clinical data for consenting/assenting eligible patients before sputum sample collection. Each patient brought the early morning sputum sample of minimum volume 4 ml in a sterile falcon tube. Before analysis, each sample was carefully homogenized and partitioned into two equal parts aseptically inside a biosafety cabinet. One of the splits was reserved for immediate detection with LED-FM and Xpert. The other half was stored for a maximum of 7 days at an appropriate temperature (2–8 °C) and then transported (in a cool box) to the National TB Reference Laboratory (NTRL), Addis Ababa, for mycobacterium culture on LJ solid media, cZN microscopy and drug sensitivity test (DST). All laboratory experiments were done following the GLI mycobacteriology laboratory manual [13].

### LED-FM

A smear was prepared on new and clean frosted slides from a sputum sample and stained with the staining reagents, auramine O stain (0.1% auramine O, 0.5% acid alcohol and 0.5% potassium permanganate) and examined by the LED-FM (Primo Star iLED, Carl Zeiss Micro Imaging, Göttingen, Germany).

### Xpert MTB/RIF® assay

The sputum samples were treated with sample reagent (SR) containing NaOH and isopropanol provided as per the manufacturer’s instruction [14]. The SR is added using a 2:1 ratio of the sputum sample, homogenized and incubated for 15 min at room temperature. From the treated samples 2 ml were transferred into multi-chambered plastic cartridge preloaded with liquid buffers and lyophilized reagent beads necessary for sample processing, deoxyribonucleic acid (DNA) extraction and hemi-nested real-time polymerase chain reaction (RT-PCR). The cartridge was loaded into the Xpert machine (GeneXpert®Dx System version 4.4a, Cepheid Company, 904 Caribbean Drive, CA 940889, USA), and an automatic process completes the remaining assay steps. The results were visualized and printable in the view results window.

### cZN smear

Specimens that were processed for culture by decontaminating using NALC-NaOH (4%) and concentrated by centrifugation at 3000×g for 15 min and decanting the supernatants were used for the cZN microscopy. Smears were prepared from the sediment on new and clean frosted slide. After the smears were well dried, ZN staining (1% carbol fucshin, 3% acid alcohol and 0.1% methylene blue) was performed and examined by LM.

Smear-positivity was determined as per the WHO definition which states that presence of at least one acid fast bacilli (AFB+) in at least one sputum sample including scanty smears [http://www.who.int/tb/laboratory/reduction_of_smears.pdf. Accessed 24 April 2017]. To declare a smear positive either LED or cZN was enough in this study.

### Mycobacterium culture on LJ medium

As the samples arrived at NTRL, the sputum samples were decontaminated using N-acetyl-L-cysteine (NALC)-sodium hydroxide NaOH (4%). After concentration by centrifugation at 3000 x g for 15 min, the sediment was re-suspended with 2 ml of 0.5 M phosphate buffer (pH 6.8) and inoculated into LJ medium and incubated at 37 °C for up to 8 weeks to declare culture-negative. Each LJ culture positive isolate was stored in NALC-tube labeled with study ID containing the storage media of mycobacterium and stored in a deep freeze refrigerator.

When the stored isolates were needed for DST, they were removed from the deep freezer and MTBC and NTM were identified using the TB Ag MPT64 Rapid Test (SD Bioline, Kyonggi-do, Korea) and inoculating the isolates on blood agar media for 48 h incubation. DST procedures were carried out on confirmed mycobacterium culture-positive samples.

### DST testing for LJ culture-positive samples

The stored isolate was cultured to mycobacteria growth indicator tube (MGIT) tube to get new and fresh isolates and first-line anti-TB drugs isoniazid (INH) and rifampicin (RIF) were tested on positive MGIT cultures. Lyophilized INH and RIF drug vials in BACTEC™ MGIT™ 960 SIRE kit were prepared (reconstituted) as per the manufacturer’s manual. Three 7 ml MGIT tubes per sample were labeled for each test isolate with GC (growth control), INH and RIF. Aseptically 0.8 ml of BACTEC™ MGIT™ 960 SIRE Supplement (provided in the SIRE Kit) was added to each tube. Hundred microliters of the reconstituted INH and RIF drugs was added to the labeled tubes for INH and RIF aseptically. The final concentration of INH and RIF drugs in the test tubes were 0.1 μg/ml and 1.0 μg/ml respectively and mycobacterium suspension were inoculated and then incubated. All procedures were carried out inside a biological safety cabinet (BSC) by using full personal protective equipment.

### Quality assurance and data collection

Sputum samples and questionnaire data were collected by trained laboratory personnel. Sputum samples were collected in sterile and leak-proof containers (falcon tubes). Samples for culture were transported to NTBL by ice pack in a cool box and performed on the same day of transportation. All laboratory tests were performed using standard operational procedures. All reagents and chemicals were of analytical grade from reputable companies. BSC was used during sample preparation including initial homogenization and splitting, culture inoculation and DST setting to ensure safety and avoid risk of contamination.

### Statistical data analysis

Socio-demographic and laboratory data were entered into SPSS version 20 (IBM SPSS) and analyzed. Sensitivity, specificity, positive and negative predicative values at 95% confidence interval (CI) of LED-FM and Xpert were calculated and compared against the gold standard LJ culture. The chi-squared test was used to test statistical significance for differences between values. All statistical tests were considered significant if the two sided *p*-value (p) was <0.05.

## Results

### Socio-demographic and clinical characteristics

From a total of 362 presumptive TB patients enrolled during the study period, majority (56.6%) were males resulting in a male to female ratio of 1.2:1.0. The age of the participants ranged from 5 to 80 years with mean of 35.3. Most (46.7%) were farmers and the least daily laborers (5.3%). Over 77.7% were able to read and write with most having knowledge about TB transmission. One hundred thirty one (39.5%) had self-reported history of previous TB medication.

Most (90.4%) of the participants knew their HIV status. Among these, 31.9% were HIV-infected. Occupationally, 42.3% of HIV positives were government employees, 20.1% farmers, 52.5% merchants, 61.1% daily laborers, 14.0% students, 55.0% housewives and 25.0% others (pensioners, prisoners, etc.).

### Detection rates

**Fig. 1:**
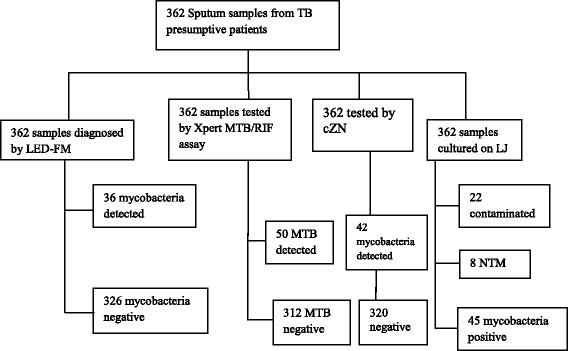
Flow chart showing series of diagnosis in the recruitment procedures and results

In total, 362 samples were successfully analyzed by LED-FM and Xpert (Fig. 1). But only 340 were cultured and declared positive or negative for the tubercle bacilli, the rest 22 were found contaminated and discarded. Eight of the 340 were found to be NTM.

Some samples were positive by one method and not by the other(s) and vice-versa. The number of samples positive by the respective methods singly and in combination is indicated in Table 1. Overall, 53 samples were found to be positive for the tubercle bacilli by at least one of the detection methods used and 35 were positive by all the e methods including culture. Specifically, 36(9.9%) samples were positive by LED-FM, 42(11.6%) by cZN and 50(13.8%) by Xpert. Xpert displays a semi-quantitative result as ‘very low’, ‘low’, ‘medium’, and ‘high’ for its positive cases. Accordingly, 7(14.0%) samples had ‘high’, 20(40.0%) ‘medium’, 16(32.0%) ‘low’ and 7(14.0%) ‘very low’ semi-quantitative results.

**Table 1:** Positivity of sputum samples analyzed by LED-FM, cZN, Xpert and LJ culture singly and in combination. PPV: positive predictive value, NPV: negative predictive value, CI: confidence interval, AIDS: acquired immunodeficiency syndrome**,** HIV: human immunodeficiency virus

		LED-FM (*n* = 362)		Xpert (*n* = 362)		cZN (*n* = 362)		LJ culture (*n* = 332)	
		positive	negative	positive	negative	positive	negative	positive	negative
LED-FM	positive (36)			36	0	36	0	35	0
	negative			14	312	6	320	10	287
Xpert	positive(50)	36	14			42	8	42	6
	negative	0	312			0	312	3	281
cZN	positive (42)	36	6	42	0			37	3
	negative	0	320	8	312			8	284
LJ culture	positive	35	10	42	3	37	8		
	negative	0	287	6	281	3	284		

TB cases detected among HIV-positives were 54.5% (6/11) by LED-FM, 63.6% (7/11) by cZN, 90.9% (10/11) by Xpert and culture 100% (11/11). Among HIV-negatives, 81.3% (26/32), 73.7% (28/38), 78.8% (26/33), 78.8% (26/33) and 73.2% (30/41) TB cases were detected by the above four methods respectively.

### LED-FM and culture

Out of 36 LED-FM-positive samples 35 were culture-confirmed, 1 was contaminated. Proportionally there was 100% agreement between LED-FM and culture concerning the former’s specificity. However, 10 culture-confirmed TB cases were missed by the LED-FM. Thus, the sensitivity, PPV and NPV of LED-FM in comparison with the gold standard LJ culture method were respectively 77.8, 100 and 96.6% (Table 2).

**Table 2:** Performance of LED-FM for detection of TB against the LJ medium culture (*n* = 332)

Characteristics	Value (%)	95% CI
Sensitivity	77.8	62.9–88.8%
Specificity	100	98.7–100%
PPV	100	90.0–100%
NPV	96.6	93.9–98.4%

*PPV* positive predictive value, *NPV* negative predictive value, *CI* confidence interval

### LED-FM and cZN

All the 36 LED-FM-positive samples were positive by the cZN as well showing 100% agreement. But 6 samples that were smear-negative by the LED-FM were positive by cZN smear. Thus the sensitivity of LED-FM compared to the conventional cZN smear is 85.7%. Thus sensitivity, specificity, PPV and NPV were 85.7, 100, 100 and 98.2% respectively. The reading agreement between cZN and LED-FM was kappa value (퐾) = 0.9.

### cZN and culture

When the cZN smear was compared against LJ culture, 3 mycobacteria detected by cZN were culture-negative. But cZN missed 8 cases that were culture-positive. Sensitivity and specificity of the cZN method were, thus, 82.2, 98.9% respectively.

### Xpert and culture

While 3 culture-confirmed mycobacterium cases were missed by Xpert, 6 of 7 Xpert-positive cases were culture-negative. Two Xpert-positive cases were indeterminate by culture because of contamination. The sensitivity and specificity of the Xpert assay in comparison with LJ culture method were 93.3 and 98.0%, respectively (Table 3).

**Table 3:** Performance of Xpert assay for detection of TB against LJ culture (*n* = 332)

Characteristics	Value (%)	95% CI
Sensitivity	93.3	81.7–98.6%
Specificity	98.0	95.6–99.2%
PPV	87.5	74.8–95.3%
NPV	98.9	97.0–99.8%

*PPV* positive predictive value, *NPV* negative predictive value, *CI* confidence interval

### Xpert and LED-FM

Concerning the performance of Xpert in relation to LED-FM, 14 Xpert-positive cases were not detected by LED-FM. But all the 36(100%) smear-positives by LED-FM were also positive by Xpert.

Xpert detected 2 RIF-resistant (MDR-TB) cases which were confirmed by the phenotypic DST test. There was no statistically significant difference in mycobacterium case detection between LED-FM, Xpert and culture among HIV-infected and non-infected TB patients.

### Socio-demography and TB positivity

Most of the cases (64.4%, 29/45) belonged to the most productive age group (15–34 years). Occupation wise, the proportion of infected daily laborers (27.8%) was the highest. Among HIV positive TB presumptive (106) patients, 10.4% were TB confirmed cases but from HIV negative TB presumptive individuals (194) about 15.5% were infected with TB. No patient had TB among individuals who refused to declare their HIV status. In all cases there was no statistically significant difference in TB prevalence with respect to the different socio-demographic variables (Table 4).

**Table 4:** Culture-confirmed TB cases in different groups of the study population (*n* = 332)

Variables		Tested (no (%)	Culture-confirmed (no (%)	*p*-value
Age (years)	≤14	44(13.3)	4(9.1)	
	15–24	47(14.2)	12(25.5)	0.767
	25–34	75(22.6)	17(22.7)	
	35–44	74(22.3)	8(10.8)	
	45–54	51(15.4)	4(7.8)	
	55–64	32(9.6)	0(0.0)	
	≥65	17(5.1)	0(0.0)	
Sex	Male	188(56.6)	23(12.2)	
	Female	152(45.8)	22(14.5)	0.695
Occupation	Employed	25(7.5)	6(24.0)	
	Farmer	155(46.7)	17(10.9)	
	Merchant	57(17.2)	9(15.8)	0.630
	Daily laborer	18(5.3)	5(27.8)	
	Student	48(14.1)	4(8.3)	
	Housewife	18(5.1)	2(11.1)	
	Others	19(5.7)	2(10.5)	
Education	Primary	193(58.1)	26 (13.5)	
	Secondary	48(14.5)	8(16.7)	0.099
	Tertiary	17(5.1)	5(29.4)	
	Uneducated	82(24.7)	6(7.3)	
HIV/AIDS status	Positive	106(31.9)	11(10.4)	
	Negative	194(58.4)	30(15.5)	
	Unwilling to respond	7(2.1)	0(0.0)	0.327
	Never tested	33(9.9)	4(12.1)	
Previous TB treatment	Yes	131(39.5)	12(9.2)	0.920
	No	209(62.9)	33(15.8)	

*AIDS acquired immunodeficiency syndrome**,** *HIV* human immunodeficiency virus

## Discussion

The 13.6% TB culture-positive rate found in this study is comparable to previous reports from various settings of Ethiopia [15]. But it was lower compared to 16.9% [16] and 25.5% [17] rates reported by other investigators. The prevalence is higher than WHO estimates for Ethiopia [1]. TB is a common public health concern in Ethiopia. Its prevalence is expected to be higher than normally reported. This is mainly because of paucity of nationwide representative incidence and prevalence data due to diagnostic challenges in most rural areas of the country. But, various prevalence data emerging from different settings are hard to compare for reasons related to the variability of such studies in their design and methods used.

Majority (56.6%) of TB presumptive patients were males. But the proportion of confirmed TB cases was higher among females (14.5%) than males (12.2%) although the difference was not statistically significant. Perhaps males had better treatment-seeking behavior and/or financial capability to travel to health-facilities. The higher proportion of female TB patients is similar to studies elsewhere in Ethiopia [16, 17]. Similar findings were reported from other countries as well [18–21]. Although reasons for such sex-based difference are not yet clear, differences in hormonal influences and production of some cytokines like tumor necrosis factors and interleukin-10 is suggested as biological factors that could enhance susceptibility of females to TB [19, 22, 23]. In other settings, a male preponderance has also been reported [24, 25].

Twenty nine (23.8%) culture-positive cases belonged to age-group 15–34 years, and these constituted 64.4% of all culture-confirmed cases. This shows the impact of the disease on this particular age group. Similarly, this age category had the highest burden in previous reports from Ethiopia [24–27] and beyond [28, 29].

Of the patients with known HIV status, about one-third was HIV-infected showing the sustained public health impact of the virus. Among these, 11(10.4%) had culture-confirmed active TB. But, this is lower compared to the proportion of TB cases (15.5%) among HIV-negatives. The lower proportion of TB cases among HIV-infected patients may be explained by the fact that this subpopulation has better health-oriented education and awareness. Probably these patients take their ART drugs effectively and get better feeding.

From culture-confirmed TB cases, 11(24.4%) were HIV co-infected. Of these 11 samples, only 6 were positive by LED-FM. But Xpert has detected all of the samples from HIV-positive cases except one. This demonstrates the diagnostic accuracy of Xpert for detection of TB among HIV-co-infected cases. Over one-third (38.5%) of the patients had history of previous treatment for TB indicating the recurring burden of the disease in the population. This study confirmed TB among 12(9.2%) of these patients. This lower finding compared to that among patients without history of previous TB treatment (15.8%) may show recent new infections rather than reactivation of past cases.

The semi-quantitative results of Xpert indicated the assay’s ability to detect a low mycobacterial load very well. Nevertheless, from 7 very-low Xpert semi-quantitative results, 6 were culture-negative. These might be the dead bacteria or due to very low bacteria in the sample, it was possibly missed during inoculation on the LJ culture tube. Also, mycobacteria were not detected by LED-FM from all the 7 very-low Xpert positive semi-quantitative results.

However, the 6 Xpert-positive but culture-negative samples undercut Xpert specificity. Xpert might have been “false-positive”. Initially sputum specimens were split inside a biosafety cabinet and the risk of specimen-to-specimen cross contamination was minimized. By its nature Xpert is less prone to contamination. Rather other factors such as prior TB treatment could have influenced Xpert specificity as it can detect DNA from nonviable, nonintact bacilli. This is the well-known limitation of molecular tests in general. The earlier a molecular test is used during effective treatment, the more chance it will pick up DNA from dead bacilli. Molecular tests are short of distinguishing between new cases and old treated infections or monitoring of treatment response.

When evaluated with serial sputum samples obtained over 26 weeks from patients undergoing treatment for TB the reduction in positivity rates with Xpert were slower than those with the standard methods [30]. Xpert may remain falsely positive, despite existing filtering system for dead mycobacteria, quite beyond the standard 6 months treatment period. This implies risk of false-positive in patients with previous treatment history, e.g. in past 12 months, though more studies are required to adequately address the issue. In this study among suspects having prior TB treatment, it was unclear when, 12 had culture-confirmed mycobacteria and 119 were culture-negative. The question is how many of these 119 belong to the 6 “Xpert-positives” which is out of the total culture-negative samples. So, the number of “Xpert-positive” but culture-negative among those with prior TB treatment history was too small to test the association between prior TB treatment and “false-positive” Xpert results. Prior TB treatment is considered a significant risk factor for Xpert false-positivity [31].

Alternatively, the single LJ culture used in this study together with it being solid might have made it less sensitive and the more sensitive Xpert might have truly detected culture-missed cases. In a recent meta-analysis it was revealed that the sensitivity and specificity of Xpert is far superior to smear microscopy as well as liquid or solid mycobacterial culture [32].

The sensitivity of cZN microscopy was not expected to be higher than that of LED-FD. Although it appears that concentration has a potential to increase smear sensitivity it is unlikely that the higher sensitivity of cZN observed in this study was solely due to the concentration step. Chattamanchi et al. 2009 [33] found no significantly higher result using concentrated sputa compared to the unprocessed. On the other hand, a significant increase in sensitivity was reported when direct smears were used (89%) rather than concentrated ones (73%) [9]. Due to inadequate generalisable evidence the WHO does not recommend sputum processing for microscopy [7].

However, 77.8% LED-FM sensitivity registered in this study was lower than what was quoted in WHO policy statement, 83.6%, [7] which was based on the systematic review by Minion et al. 2009 [4]. In fact, various studies showed that LED-FM has 10% higher sensitivity over ZN [4, 6, 34]. However, a recent comprehensive meta-analysis [35] using data between January 1, 2000 and April 1, 2014 in 11 databases reported a pooled sensitivity of 66.9% for LED-FM. Change et al. 2016 [35] suggested that study methodology factors and differences in the LED-FM procedure or device (e.g. commercial LED systems) could affect LED-FM performance. Their study showed that study-specific sensitivities varied from 40 to 83%, while specificities varied from 82 to 100% showing a high level heterogeneity among the analysed studies. A visible influence of clinical settings on the diagnostic accuracy of LED-FM and a higher variability in sensitivity associated with research/referral hospitals. The study also suggested that liquid culture reference standard was associated with higher diagnostic accuracy of LED-FM. The same study further suggested that culture reference based on more than one specimen improved the diagnostic accuracy of LED-FM.

Experience and access to appropriate and adequate training may also affect the performance estimates of LED. In a recent study from Rwanda [36] the sensitivity of LED-FM (37%) was lower than that of cZN (55.1%) in peripheral laboratories although the result was 62.5% and 58.3%, respectively, in intermediate laboratories suggesting some differences in the skills of the microscopists.

In the current study eight samples (15.1%) that grew on LJ medium were NTM. Worodria et al. 2011 [37] reported 100% specificity of 80 LJ cultures for MTBC growth. However, various studies from sub-Saharan Africa as well as other regions recorded NTM detection in LJ medium. For instance, reports from Nigeria [38], South Africa [39], Mozambique [40] and Zambia [41] reported NTM cases ranging from 14 to 23%. Indeed the frequency of NTM varies depending on the geographic location, study design, population, and sample type. In India, 2% of 6143 AFB-positive cultures were identified as NTM based on speciation by rpoB sequencing; 6 of these were found to be pulmonary cases of mixed NTM and MTBC infection [42]. A recent report showed high rates (26.3%) of NTM in Mozambican children with presumptive TB [43].

However, concerning rapid mycobacterium using MPT64 has associated limitations. In the absence of sequencing false-negative MPT64 could not be excluded. Hirano et al. 2004 [44] detected mutations within the *mpb64* gene and the test might have missed some MTBC cases. On the other hand, the same authors reported that NTM isolates testing positive with Ag MPT64 assay were also detected. Unfortunately Xpert was not performed on the 8 MPT64 negative samples (among the 45 cultures positives for mycobacteria) in this study. The existence of false-positive samples is equally possible.

Although infrequent, NTM can be associated with active TB cases such as cervicofacial lymphadenitis, followed by skin and soft tissue infections especially in children and occasionally it may induce PTB and disseminated infection in people with certain genetic disorders or HIV [45]. The most important concern of NTM, however, is that it can contribute to TB misdiagnosis complicating early detection of MTBC.

The major limitation of this study is that a single solid LJ culture was considered the gold standard. MGIT liquid culture is more sensitive than LJ and a better yield is obtained with a second sputum culture. The WHO recommended that two sputum specimens be examined in the investigation of suspected PTB. These, coupled with the aforementioned factors, might have accounted for the relatively lower LED-FM sensitivity and Xpert specificity, and overall culture yield compared to studies that recorded higher results. Yet another limitation was not Xpert testing the 8 MPT64 negative samples that were identified as “NTM”.

The 2 RIF-resistant cases detected by Xpert and DST were not from HIV-infected individuals showing that RIF-resistance is not related to TB-HIV infection. Both of the cases were from TB patients with previous history of treatment suggesting reactivation of previous latent cases rather than new infections.

## Conclusion

The Xpert assay showed full conformity with the LJ solid culture gold standard in its specificity although its sensitivity was perhaps weakened due to 6 culture-negative samples it detected. The 100% specificity of LED-FM demonstrates that the device is performing very well in the study setting although its lower sensitivity compared to the cZN needs future corroboration.

## Abbreviations

ART: Antiretroviral therapy
BSC: Biological safety cabinet
CI: Confidence interval
DS: Drug sensitivity test
GC: Growth control
HIV: Human immunodeficiency virus
INH: Isoniazid
LED**-**FM: Light-emitting diode florescent microscopy
LJ: Lowenstein-jensen
MDR: Multi-drug resistant
MGIT: Mycobacteria growth indicator tube
NTRL: National TB reference laboratory
RIF: Rifampicin
TB: Tuberculosis
WHO: World health organization
